# A Single Bout of Aerobic Exercise Increases Neuronal Extracellular Vesicle‐Derived Insulin Signaling Biomarkers in Adults With Cardiometabolic Risk

**DOI:** 10.1002/cph4.70131

**Published:** 2026-03-24

**Authors:** Steven K. Malin, Daniel J. Battillo, Michal S. Beeri, Maja Mustapic, Dimitrios Kapogiannis

**Affiliations:** ^1^ Department of Kinesiology and Health Rutgers University New Brunswick New Jersey USA; ^2^ Department of Medicine; Division of Endocrinology, Metabolism and Nutrition Rutgers University New Brunswick New Jersey USA; ^3^ New Jersey Institute for Food, Nutrition and Health Rutgers University New Brunswick New Jersey USA; ^4^ Institute of Translational Medicine and Science Rutgers University New Brunswick New Jersey USA; ^5^ Institute for Health, Health Care Policy and Aging Research Rutgers University New Brunswick New Jersey USA; ^6^ National Institute on Aging Baltimore Maryland USA

**Keywords:** Alzheimer's disease, brain insulin resistance, physical activity, type 2 diabetes

## Abstract

Exercise may lower Alzheimer's Disease and Related Dementia (ADRD) risk. While insulin has been proposed to benefit cognition, the effect of exercise on neuronal insulin signaling in humans is unclear.

**Purpose:**

We tested the hypothesis that a single bout of aerobic exercise would raise insulin signaling mediators from plasma‐derived neuronal extracellular vesicles (nEVs).

**Methods:**

Fifteen sedentary adults with obesity (12F; ~56y; ~31 kg/m^2^) completed an evening rest and acute exercise condition (70% maximal oxygen consumption (VO_2_max)) in a randomized, counterbalanced order. Following an overnight fast, plasma was collected for analysis of nEV insulin signaling biomarkers before and after intranasal insulin spray (INI, 40 IU) as well as 60 min following a 75 g oral glucose tolerance test (OGTT). Plasma glucose and insulin were also measured at 30 and 60 min during the OGTT, and total area under the curve (tAUC) was calculated.

**Results:**

Exercise tended to lower glucose tAUC_0‐150min_ (*p* = 0.08, d = 0.50), independent of insulin tAUC_0‐150min_ (*p* = 0.99, d = 0.00). Exercise increased pIR‐Tyr1162/Tyr1163 (*p* = 0.05, *η* ^2^ = 0.05), pIRS‐1‐Ser636 (*p* = 0.02, *η* ^2^ = 0.07), pAkt‐Ser473 (*p* = 0.03, *η* ^2^ = 0.06), and pTSC2‐Ser939 (*p* = 0.01, *η* ^2^ = 0.08) with medium effect sizes across blood draws, compared with the resting condition. Exercise also raised fasting and decreased pp70S6K‐Thr412 before and after the OGTT, compared with increased levels after rest during the OGTT (*p* = 0.02, *η* ^2^ = 0.10). Exercise had no effect on other insulin signaling proteins (e.g., pmTOR‐Ser2448, pGSK3β‐Ser9, etc.).

**Conclusions:**

A single bout of aerobic exercise increases some nEV‐associated insulin signaling phosphoproteins in people with cardiometabolic risk. Additional work is warranted to determine if changes in brain insulin signaling translate to lower ADRD risk.

**Clinical Trials Registration:**

NCT05853913.

## Graphical abstract

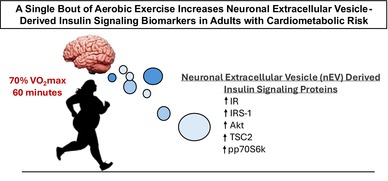

## 1 Introduction

Obesity, type 2 diabetes (T2D), and cardiovascular disease increase the risk of Alzheimer's disease (AD) and related dementias (ADRD) (Mittal and Katare 2016; Malin et al. 2022). A possible link between these diseases may relate to brain insulin resistance. Although the brain is traditionally considered insulin independent with respect to glucose utilization, insulin acts in the healthy brain to promote synaptogenesis, neuronal cell differentiation, and amyloid‐β plaque degradation as well as regulate peripheral metabolic and vascular function (Agrawal et al. 2021). These functions are impaired among individuals with obesity and T2D, who can also showcase brain hypoinsulinemia as a consequence of decreased insulin transporter expression at the blood–brain barrier (Arnold et al. 2018). Neuronal extracellular vesicles (nEVs) have emerged as a potential “liquid biopsy” of the brain since they can cross the blood brain barrier and be assessed from peripheral blood draws (Cleary et al. 2025; Manolopoulos et al. 2025). nEVs contain phosphoprotein mediators involved in the canonical insulin signaling cascade (e.g., IR, IRS‐1, and Akt) as well as overlapping cascades (e.g., pm‐TOR, p70S6K, and TSC2) with important effects on neuronal cellular metabolism, survival, and repair (Kapogiannis et al. 2019). Altered nEV insulin signaling proteins have been reported in individuals with AD, T2D, and frontotemporal dementia, even several years prior to formal diagnosis (Kapogiannis et al. 2015). Whether nEV‐associated insulin signaling mediators respond to insulin‐sensitizing treatments and interventions is an area of work requiring further investigation.

Lifestyle interventions like exercise remain foundational for cognitive health. People who adhere to exercise guidelines, particularly throughout midlife, show higher executive function and brain volume with aging (Erickson et al. 2019). How exercise mechanistically contributes to improvements and/or maintenance of cognition throughout aging is likely to be multifactorial, with several groups suggesting brain‐derived neurotrophic factor as a mediator (Malin et al. 2022). Yet a potentially novel mechanism by which exercise may foster cognition is by improving brain insulin sensitivity (Malin et al. 2022). Indeed, recent studies in people with excess weight and insulin resistance suggest that exercise training promotes insulin‐stimulated cerebral blood flow (CBF) (Kullmann et al. 2022) as well as brain glucose metabolism (Honkala et al. 2018). These functional responses are supported by rodent studies showing increased brain insulin signaling following aerobic exercise (Kang and Cho 2014). Whether the functional changes in brain insulin sensitivity in humans correspond with enhanced brain insulin signaling is unclear given the challenges with obtaining brain tissue for conventional assessments. Hence, nEVs have emerged as a second‐best surrogate platform for such assessments. To date, there is one study examining the effects of exercise on nEV insulin signaling cargo, and it was reported that 2 weeks of aerobic exercise in older adults with prediabetes increased glucose‐stimulated total Akt from nEVs (Malin, Battillo, et al. 2025). However, participants in this prior work as well as those examining CBF and brain glucose metabolism studies lost weight and/or experienced increased aerobic fitness, which may alter interpretations of exercise effects on the brain. Recently, we reported that a single bout of exercise performed in the evening increased fasting CBF the next morning in multiple brain regions, including the hippocampus, and these changes corresponded with greater intranasal insulin‐mediated CBF responses in middle‐aged to older adults with cardiometabolic risk (Malin, Shah, et al. 2025). Whether a single bout of exercise in humans, though, can increase insulin signaling as assessed by nEVs is unknown. The purpose of the current study was to examine if an acute exercise bout impacts nEV insulin signaling before and after intranasal insulin (INI) administration as well as during an OGTT among middle‐aged to older adults with obesity. We hypothesized that exercise would raise fasting nEV insulin signaling mediators in relation to insulin stimulation as demonstrated by our CBF results before (Malin, Shah, et al. 2025).

## 2 Methods

### 2.1 Study Design and Participants

These were the same 15 participants enrolled in prior studies examining the single bout effects of exercise on CBF and cognition (Malin, Shah, et al. 2025). Herein, we focus outcomes on nEV‐associated biomarker outcomes. Participants underwent a randomized, counterbalanced study and were recruited from the New Brunswick, NJ area via paper advertisements, social media, and/or electronic medical records. Individuals were excluded if they were smoking, physically active as evident by self‐report (> 150 min/week of moderate intensity exercise), weight unstable > 2 kg over the last 3 months, taking insulin for glycemic control, had mild cognitive impairment (MCI)/dementia as operationalized via the Montreal Cognitive Assessment (MoCA, < 25 score), and had chronic disease (e.g., renal, hepatic, cardiovascular, etc.). A 12‐lead electrocardiogram (EKG) was completed at rest and during maximal exercise to assess heart rhythm. A physician evaluated blood biochemistries and EKGs in conjunction with a physical exam to ensure eligibility. The study protocol conforms to the Declaration of Helsinki and was approved by Rutgers University Institutional Review Board (IRB #: 2022001842). People provided written and verbal consent. The overall study was registered on Clinicaltrials.gov (NCT # 05853913).

### 2.2 Body Weight, Resting Metabolic Rate, and Cardiorespiratory Fitness

Total body weight was recorded using a digital scale and waist circumference was measured in duplicate using a tape measure. Height was measured to the nearest 0.1 cm using a stadiometer. Resting metabolic rate (RMR) was determined with participants lying supine following approximately 20 min of rest using a ventilated hood (COSMED Quark, Chicago, IL). Breath samples were assessed for 15 min using indirect calorimetry and data were averaged over the last 5 min for analysis. Participants rested in a semi‐supine position. Cardiorespiratory fitness (VO_2_max) was assessed on a treadmill using indirect calorimetry as previously done by our laboratory (Heiston et al. 2021).

### 2.3 Intervention Visits

**FIGURE 1:**
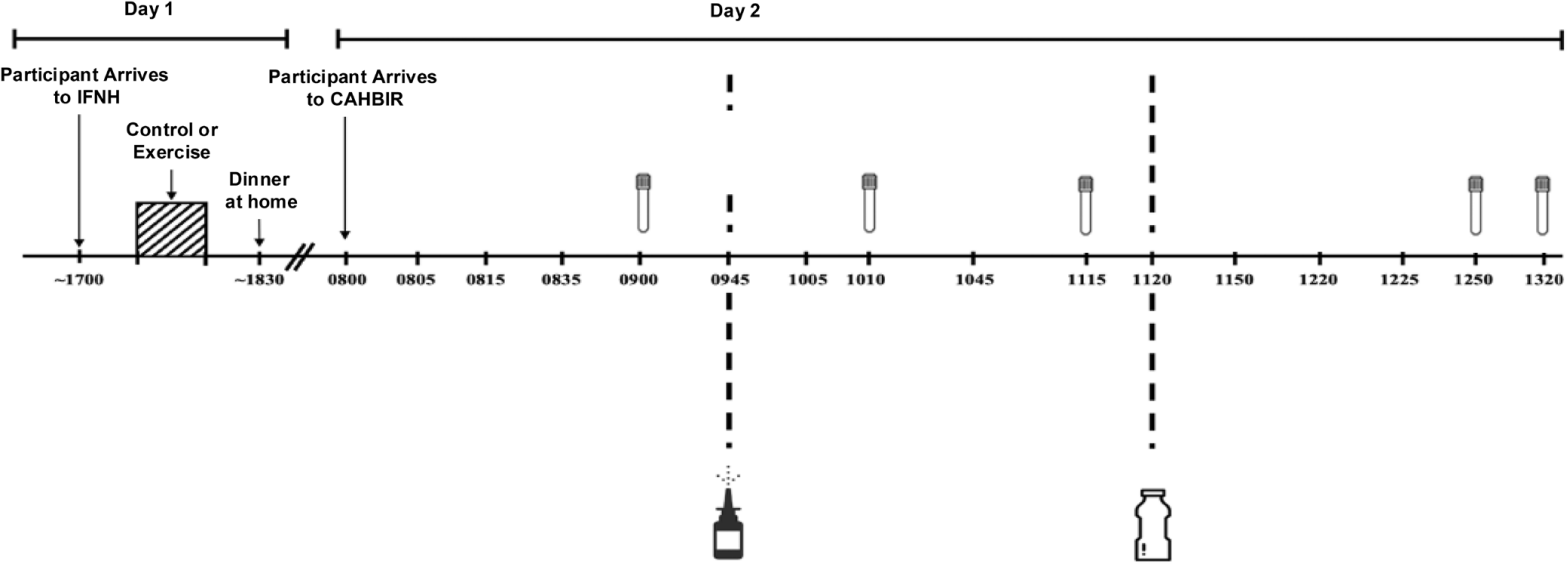
Study design.

A detailed description of our interventions has been previously reported (Malin, Shah, et al. 2025). In short, participants completed a resting (control) or exercise condition the night prior to testing in a randomized, counterbalanced order (Figure 1). This protocol was selected based on our prior work showing improved vascular insulin sensitivity after one exercise bout (Heiston et al. 2021). The resting condition consisted of 60 min of supervised seated rest and was completed at the same time as exercise to ensure minimal participant movement or activity. The exercise condition consisted of 5 min of resting indirect calorimetry to establish baseline measures followed by a 5 min warmup (45% VO_2_max) and 60 min of treadmill walking at 70% VO_2_max. Participants were provided standardized meals (~55% carbohydrate, 30% fat, and 15% protein) during the day of each intervention visit and instructed to eat their dinner meal at home following completion of the condition visit. Participants were also instructed to refrain from caffeine, alcohol, and medication for 24 h prior to clinical testing.

### 2.4 Clinical Testing

Participants arrived at the Center for Advanced Human Brain Imaging Research (CAHBIR) after a 10–12 h fast and approximately 15 h following the experimental conditions (Figure 1). Upon arrival, participants were placed in an upright chair in a quiet room for 5 min, and an IV line was placed to obtain blood samples for fasting glucose, insulin, and nEVs. Thereafter, participants were moved to the MRI room as previously described (Malin, Shah, et al. 2025) to measure CBF. Following scanning, participants were moved outside the MRI room and were administered INI at a dose of 40 IU of human insulin (0.4 mL, Humulin, Eli Lilly & Co., Indianapolis, IN). Single puffs of 0.1 mL occurred 4 times (twice each nostril) over 2 min via the VianaseTM electronic atomizers (Kurve Technology Inc., Lynnwood, WA). After 30 min, blood samples for glucose, insulin, and nEVs were obtained again. nEV was selected to be examined at 30 min post INI given prior work we described and performed suggested insulin optimally stimulates CBF (Malin, Shah, et al. 2025). Thus, we rationalized this may coincide with activating nEV insulin signaling. Blood for glucose and insulin were also obtained after imaging (i.e., at 90 min), and then individuals underwent a 75 g oral glucose tolerance test (OGTT). Blood samples were obtained at 30 and 60 min after glucose ingestion (i.e., 120 and 150 min) to assess glucose and insulin, and at 60 min (i.e., 150 min) to obtain nEVs. This time‐point of 60 min post OGTT was selected based on our prior work showing changes in Akt from nEVs after 2 weeks of exercise (Malin, Battillo, et al. 2025).

### 2.5 nEV Isolation

nEVs were isolated following the immunoaffinity capture methodology targeting the transmembrane neuronal markers: L1 Cell Adhesion Molecule (L1CAM), Growth‐Associated Protein (GAP43), and Neuroligin 3 (NRGN3). A detailed characterization of L1CAM+/GAP43+/NLGN3+ nEVs has been recently published (Kapogiannis et al. 2024). There, we provide a detailed characterization of nEVs by multiple methodologies (Nanoparticle Tracking Analysis, Electron Microscopy, ELISAs for plasma soluble proteins/contaminants, fluorescent immunolabeling for neuronal markers, flow cytometry analysis for neuronal markers). Here in short, volumes of all reagents were normalized to the initial plasma volume throughout the isolation protocol, thus ensuring that biomarker outcomes were not confounded by nEV recovery. Reagent volumes are expressed per 500 μL of plasma. First, plasma was spun 12,000 × *g* for 10 min at RT. SmartSEC plate (Systems Biosciences, cat. no. SSEC096A‐1) was prepared according to the manufacturer's instructions. SmartSEC plate was washed twice with 500 μL of SmartSEC isolation buffer and spun 1 min at 500 × *g*. After washing, we applied 500 μL of plasma to the washed resin in each well. SmartSEC plate was sealed and incubated for 30 min at RT. Following incubation, SmartSEC plate was spun 2 min at 500 × *g*, which produced the first fraction of 500 μL. Afterwards, the plate was washed again with a SmartSEC isolation buffer (500 μL), which produced fraction 2. Both fractions were combined and subjected to immunoprecipitation. To each tube containing 1 mL of crude EVs, we added a cocktail of antibodies containing 3 μg L1CAM‐biotin (clone 5G3, cat. no. ab 13171982) and 6 μg ExoSORT antibodies. ExoSORT antibodies are a cocktail of GAP43 and NLGN3 antibodies at the concentration of 1 mg/mL purchased from Neurodex, Natick, MA (these are proprietary antibodies and no clone information is available). Crude EVs with added biotinylated antibodies were incubated with rotation for 2 h at RT. After 2 h, 75 μL of magnetic beads (MyOne T1 Dynabeads, ThermoFisher Scientific, cat. no. 65601) were added to the tubes and additionally incubated for 1 h at RT. Following incubation, the tubes were set at the magnetic rack and washed twice with isolation buffer (1% w/v BSA with 0.1% Tween‐20 in PBS). After the wash, nEVs (neuronal enriched EVs) were eluted from the magnetic beads using 115 μL of 0.1 M glycine (stock solution 1 M, pH 2.7, cat. no. 24,074‐500; Polysciences Inc., Warrington, PA, USA). To neutralize pH, 10 μL of 1 M Tris‐HCl (pH 8.0, cat. no. CAS1185‐53‐1; Fisher Scientific, Waltham, MA, USA) was added to nEVs. The produced volume was subjected to lysis via addition of 187.5 μL of mammalian protein extraction reagent (M‐PER) lysis buffer (cat. no. 78501; Thermo Scientific) supplemented with 1.7X protease/phosphatase inhibitor cocktail, incubated 30 min at 4°C, and subsequently stored at −80°C.

### 2.6 Biomarker Determination in nEV Lysates

The MILLIPLEX Akt/mTOR Phosphoprotein Magnetic Bead 11‐Plex Kit—Cell Signaling Multiplex Assay (Millipore Sigma, Burlington, MA, USA) was used to measure pIR‐Tyr1162/Tyr1163, pIRS‐1‐Ser636, pAkt‐Ser473, pGSK3α‐Ser21, pGSK3β‐Ser9, pIGF1R‐Tyr1135/Tyr1136, pmTOR‐Ser2448, pp70S6K‐Thr412, pPTEN‐Ser380, pRPS6‐Ser235/Ser236, and pTSC2‐Ser939. nEV samples were run in duplicate with input based on experiments identifying the optimal sample dilution resulting in signals within the dynamic range of each assay. Standard curve equation for the ELISA was determined using the four‐parameter logistic (4‐PL) regression; the MILLIPLEX lacked standards, but included positive and negative controls (cell lysate and nEV matrix, respectively); therefore, arbitrary units are reported. Quality control was further based on CV of duplicates (values > 20% were excluded) and calculation of limit of detection (LOD) with values below LOD being excluded.

### 2.7 Biochemical Analyses

Plasma glucose was collected in vacutainers containing sodium fluoride, while plasma insulin was collected in EDTA vacutainers containing aprotinin and centrifuged at 4°C for 10 min at 3000 RPM. All bloods were frozen at −80°C until analysis. Plasma glucose was analyzed via the glucose oxidase method (YSI Instruments 2500, Yellow Springs, OH, USA). Plasma insulin was analyzed via enzyme‐linked immunosorbent assays (ALPCO, Salem, NH, USA).

### 2.8 Statistical Analysis

This was a secondary analysis to test the efficacy of acute exercise on fasting, INI‐stimulated, and post‐OGTT nEV insulin signaling biomarkers. Data were analyzed using SPSS (IBM, V. 28.0, Armonk NY, USA). Normality was assessed via the Shapiro–Wilk test, and non‐normally distributed data were log transformed. A repeated measures analysis of variance (ANOVA) was used to assess differences between the resting and exercise conditions. The Greenhouse–Geisser correction was applied to adjust for violations of sphericity, if applicable. Because fasting nEV values were quantifiably higher after exercise, they were included as covariates in the ANOVA model for outcomes initially identified as statistically significant to assess effects of these fasting changes on exercise induced effects over time as secondary analysis. Pairwise comparisons were conducted using the Benjamini‐Hochberg procedure to control for the false discovery rate (FDR). Additionally, fasting and tAUC values were calculated and compared between conditions via a two‐tailed paired *t*‐test. Effect sizes were calculated to assess the physiological relevance among group differences, with Cohen's d for paired *t*‐tests interpreted as small (d = 0.2), medium (d = 0.5), and large (d = 0.8) and partial eta squared (*η* ^2^) for repeated measures ANOVAs interpreted as small (*η* ^2^ = 0.01), medium (*η* ^2^ = 0.06), and large (*η* ^2^ = 0.14). Given that we previously reported rises in fasting CBF following a single bout of exercise (Malin, Shah, et al. 2025), we sought to assess if changes in nEVs were associated with changes in fasting CBF by way of Pearson Correlations. Significance was accepted as *p* ≤ 0.05, and data are presented as mean ± SEM.

## 3 Results

### 3.1 Participant Characteristics

As individual demographics were previously reported, here we provide average descriptives to help with interpretations (Malin, Shah, et al. 2025). In brief, people were cognitively unimpaired (MOCA: ~28.2 a.u.), middle‐aged (~56 years), sedentary (VO_2_max ~23 mL/kg/min) with excess body weight (BMI: ~31.8 kg/m^2^), and had impaired glucose tolerance (HbA1c, ~5.8%) as previously described (Malin, Shah, et al. 2025). All participants performed a single bout of exercise at moderate to high intensity (~69.4% VO_2_max; with an RPE of 12.4 ± 0.4 a.u.).

### 3.2 Glucose and Insulin Concentrations

There were no differences in fasting plasma glucose or insulin between rest and exercise conditions as previously described (Malin, Shah, et al. 2025). Further, plasma glucose and insulin did not change 30 or 90 min following INI in either condition (Malin, Shah, et al. 2025). However, plasma glucose tAUC_0–150min_ tended to be lower, albeit it did not reach statistical significance, following exercise (*p* = 0.08, d = 0.50), despite no difference in plasma insulin tAUC_0–150min_ between conditions (*p* = 0.99, d = 0.00; Table 1).

**TABLE 1:** Fasting and tAUC of glucose and insulin.

	Rest	Exercise	*p*	Cohen's d
Glucose				
tAUC_0‐OGTT150min_	17106.6 ± 668.6	16677.6 ± 600.8	0.08	0.50
Insulin				
tAUC_0‐OGTT150min_	4048.8 ± 483.6	4044.6 ± 516.4	0.99	0.00

*Note:* Data are mean ± SEM. 0 min = pre intranasal insulin or fasting. OGTT 150 min = 60 min after 75 g glucose ingestion.

Abbreviation: tAUC, total area under the curve.

### 3.3 nEV Insulin Signaling Biomarkers

**FIGURE 2:**
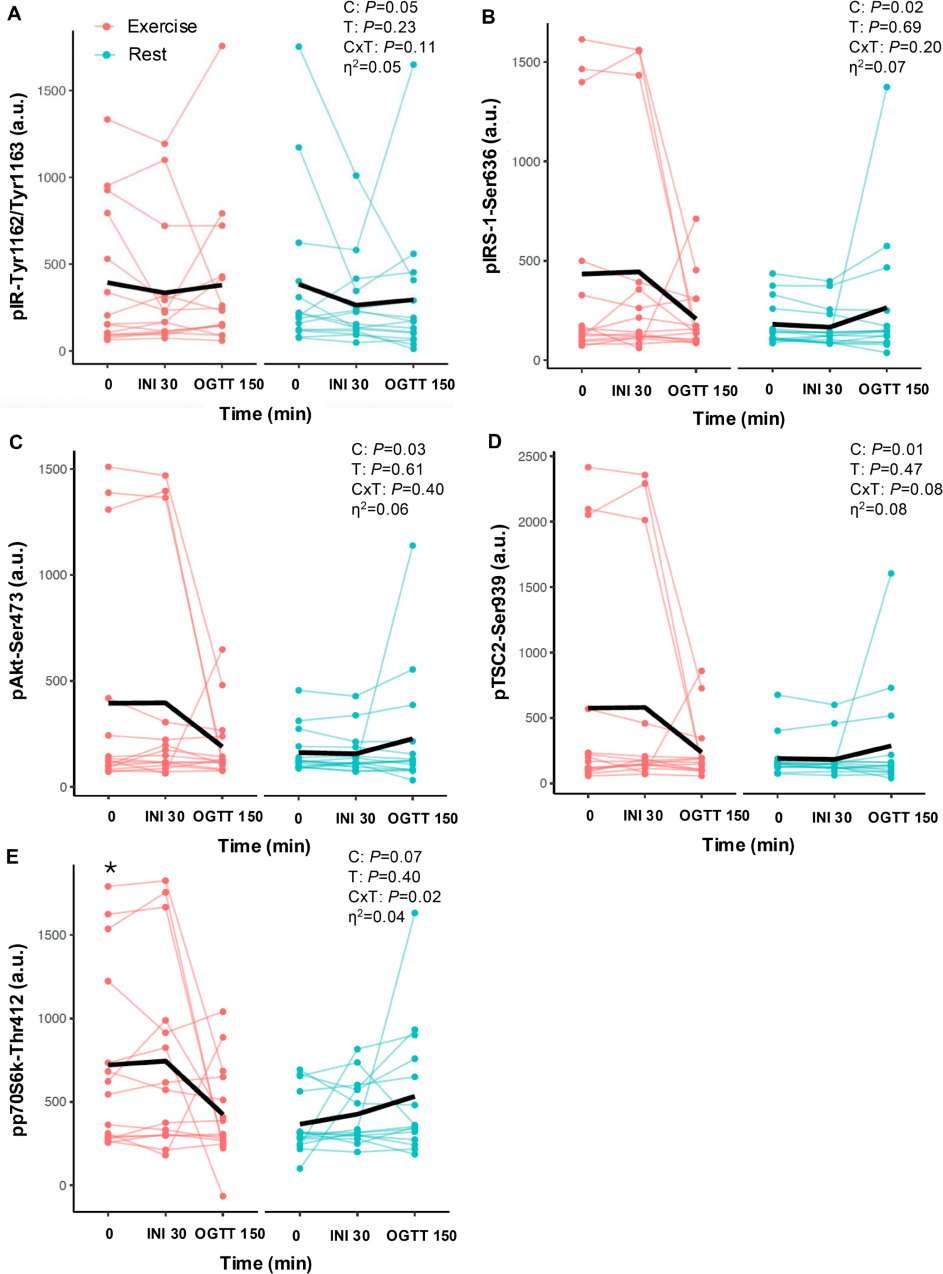
Effect of intranasal insulin and an oral glucose tolerance test on pIR‐Tyr1162/Try1163 (A), pIRS‐1‐Ser636 (B), pAkt‐Ser473 (C), pTSC2‐Ser939 (D), and pp70S6K‐Thr412 (E) concentrations. Data are mean (black bar) and individual responses. Two‐way condition (C) by time (T) repeated measures ANOVAs were performed without adjustment to determine effects between conditions across fasting (0 min), intranasal insulin (INI, 30 min), and oral glucose tolerance (150 min or 60 min after glucose ingestion). Data were log‐transformed for analysis. Effect sizes (*η* ^2^) are reported for condition effects.

The acute exercise bout increased pIR‐Tyr1162/Tyr1163 (condition effect; *p* = 0.05, *η* ^2^ = 0.05; Figure 2) and pIRS‐1‐Ser636 (condition effect; *p* = 0.02, *η* ^2^ = 0.07; Figure 2) compared with the resting condition. After adjusting pIR‐Tyr1162/Tyr1163 and pIRS‐1‐Ser6 for fasting changes after exercise, it was observed that the condition effect was no longer statistically significant (*p* = 0.21, *η* ^2^ = 0.05 and *p* = 0.20, *η* ^2^ = 0.02, respectively). Further, exercise tended to increase both pAkt‐Ser473 (condition effect; *p* = 0.03, *η* ^2^ = 0.06; Figure 2) and pTSC2‐Ser939 (condition effect; *p* = 0.01, *η* ^2^ = 0.08; Figure 2) with medium effect sizes. After adjusting pAkt‐Ser473 and pTSC2‐Ser939 for fasting changes after exercise, it was observed that the condition effect was no longer statistically significant (*p* = 0.28, *η* ^2^ = 0.02 and *p* = 0.46, *η* ^2^ < 0.01, respectively). Exercise also tended to raise pp70S6K‐Thr412 overall (condition effect, *p* = 0.07, *η* ^2^ = 0.04) compared with rest. Interestingly, exercise decreased pp70S6K‐Thr412 during the OGTT, while it increased after rest (condition × time interaction; *p* = 0.02, *η* ^2^ = 0.10; Figure 2). Specifically, fasting pp70S6K‐Thr412 was higher after exercise than the resting condition (*p* = 0.05). After adjusting pp70S6K‐Thr412 for fasting changes after exercise, it was observed that the condition effect was no longer statistically different (*p* = 0.28, *η* ^2^ = 0.02). However, the pp70S6K‐Thr412 interaction remained (condition × time interaction; *p* = 0.03, *η* ^2^ = 0.10). No exercise effect was observed for other insulin signaling proteins (Table 2).

**TABLE 2:** Fasting, intranasal insulin‐stimulated, and oral glucose‐stimulated nEV insulin signaling proteins.

	Rest			Exercise			C	T	C×T	*η* ^2^
	0 min	INI 30 min	OGTT 150 min	0 min	INI 30 min	OGTT 150 min				
pPTEN‐Ser380 (a.u.)	579.7 ± 145.4	498.6 ± 124.3	505.0 ± 143.5	570.8 ± 138.0	432.1 ± 87.8	542.9 ± 153.1	0.84	0.29	0.39	0.03
pIGF1R‐Tyr1135/Tyr1136 (a.u.)	310.0 ± 43.1	294.8 ± 36.7	281.7 ± 37.8	289.5 ± 41.8	276.6 ± 35.2	289.6 ± 35.0	0.35	0.98	0.13	0.06
pmTOR‐Ser2448 (a.u.)	217.6 ± 30.7	189.4 ± 19.0	196.4 ± 26.7	201.6 ± 26.1	187.3 ± 17.5	194.1 ± 18.2	0.91	0.66	0.71	0.01
pRPS6‐Ser235/Ser236 (a.u.)	216.6 ± 46.4	175.5 ± 29.3	179.9 ± 33.6	184.9 ± 35.0	161.2 ± 16.5	172.9 ± 26.1	0.56	0.61	0.65	0.01
pGSK3α‐Ser21 (a.u.)	138.7 ± 16.7	129.3 ± 9.9	127.4 ± 13.7	132.8 ± 13.6	123.8 ± 7.5	133.6 ± 12.9	0.92	0.85	0.63	0.01
pGSK3β‐Ser9 (a.u.)	149.2 ± 27.5	120.3 ± 16.4	118.9 ± 18.2	131.7 ± 19.1	115.1 ± 11.9	121.7 ± 14.8	0.97	0.25	0.63	0.01

*Note:* Data are mean ± SEM. Data presented were analyzed by repeated measures ANOVA without adjustment. Data were log‐transformed for analysis. C = Condition, T = time. *η* ^2^ is displayed for C×T interaction. OGTT 150 min = 60 min after glucose drink.

Abbreviations: GSK3α, glycogen synthase kinase 3 alpha; GSK3β, glycogen synthase kinase 3 beta; IGF1R, insulin‐like growth factor 1 receptor; mTOR, mammalian target of rapamycin; PTEN, phosphatase and tensin homolog; RPS6, ribosomal protein S6.

### 3.4 Correlations

**FIGURE 3:**
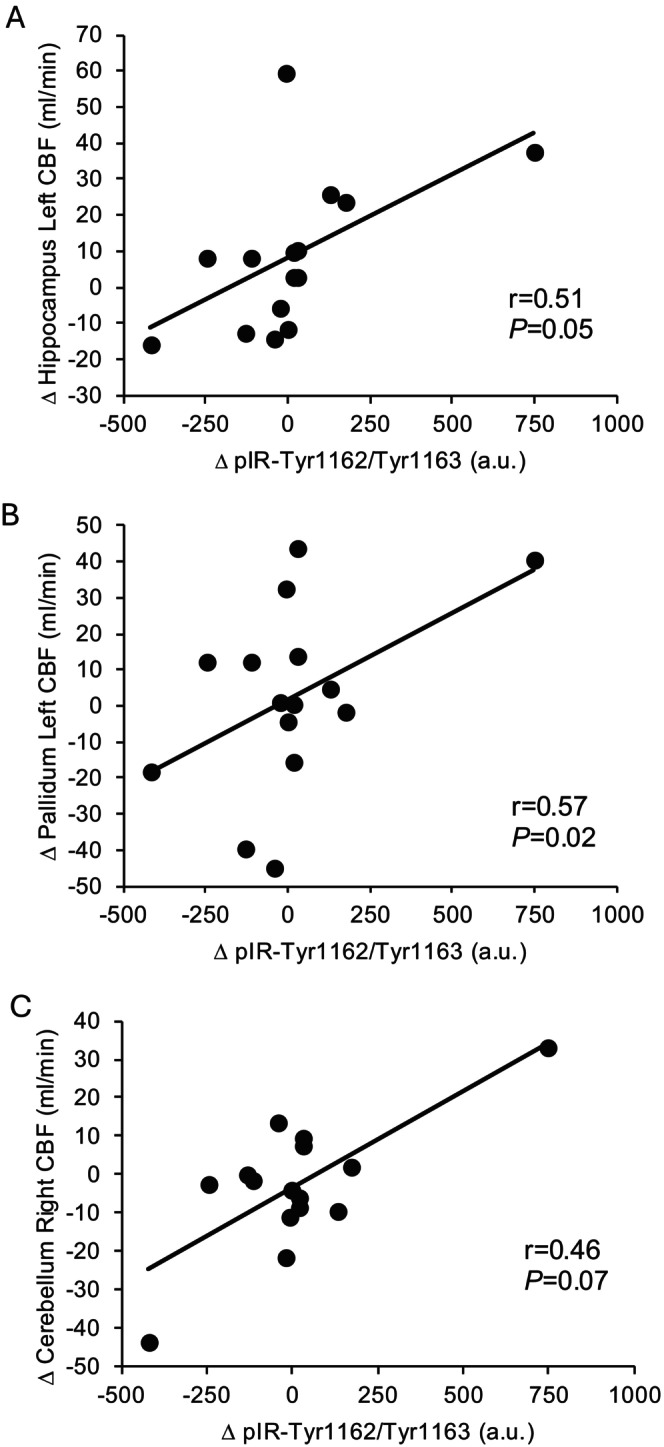
Correlation of nEV pIR‐Tyr1162/Try1163 changes (∆) after exercise with changes in cerebral blood flow (CBF). nEV data were log transformed for analysis, but raw data are presented for ease of interpretation.

Increased fasting pIR‐Tyr1162/Tyr1163 after exercise (Figure 3) was significantly associated with increased fasting left hemisphere hippocampus CBF (*r* = 0.51, *p* = 0.05) and pallidum CBF (*r* = 0.57, *p* = 0.02) as well as a trend with right hemisphere cerebellum CBF (*r* = 0.46, *p* = 0.07).

## 4 Discussion

Insulin action in the brain has been implicated in neuronal cell survival, growth, and metabolism as well as neuroprotection by, in part, reducing amyloid‐β deposition and tau hyperphosphorylation (Milstein and Ferris 2021). In fact, nearly 20 years ago it was suggested that AD/ADRD may be tied to brain insulin resistance as evident by abnormalities in the expression of phosphorylated insulin signaling proteins (Arnold et al. 2018; Gabbouj et al. 2019). In subsequent years, abnormal changes in phosphorylation levels of IRS‐1‐Akt‐mTOR and GSK3α/β pathways as well as IGFR have been noted in post‐mortem brains using ex vivo insulin stimulation, particularly from hippocampal tissue (Talbot et al. 2012). This is clinically relevant since blunted insulin signaling reduces activation of GSK3α/β in neurons and relates to increased tau phosphorylation (Talbot et al. 2012). The primary finding from this study is that a single bout of aerobic exercise performed in the evening increased some nEV‐associated insulin signaling biomarkers the next morning compared with rest in adults with cardiometabolic risk. Notably, exercise in the present study increased pIR‐Tyr1162/Tyr1163, pIRS‐1‐Ser636, pAkt‐Ser473, and pTSC2‐Ser939 with medium effect sizes. We also find that pp70S6K‐Thr412 increased under fasting states and decreased after glucose ingestion after exercise when compared with the resting condition with large effect sizes. Together, these findings suggest that a single bout of moderate to vigorous exercise may enhance some aspects of the insulin signaling pathways in the brain. These findings are not only similar to prior work from our group demonstrating that 2 weeks of aerobic exercise increased nEV‐associated total Akt after glucose ingestion (Malin, Battillo, et al. 2025), but are also consistent with other recent work by our lab showing that exercise raises CBF in the hippocampus and putamen, brain areas traditionally considered as responsive to insulin (Malin, Shah, et al. 2025). In fact, here we expand this latter work and show that rises in fasting nEV derived pIR‐Tyr1162/Tyr1163 were modestly associated with increases in fasting hippocampus, pallidum, and cerebellum CBF. Collectively, these findings align with other work in the literature suggesting that moderate to vigorous exercise training for 8 weeks can raise CBF following 160 INI administration in young adults with obesity (Kullmann et al. 2022). Moreover, 2 weeks of sprint interval training, but not moderate continuous training, decreased insulin‐stimulated glucose uptake in cortical gray matter as assessed by positron emission tomography (PET) in insulin resistant, middle‐aged adults (Honkala et al. 2018). Although a decrease in insulin‐stimulated brain glucose metabolism may seem counterintuitive, it may be attributable to improved fasting brain glucose metabolism, dampening further insulin‐stimulated changes. Therefore, the present study both aligns with and expands upon work examining CBF and brain glucose metabolism responses to insulin by understanding how a single bout of moderate to high intensity exercise promotes brain insulin signaling. Additional work here is warranted to understand if improved brain insulin signaling translates into preventing and/or delaying the onset of ADRD.

We did not find any changes in pPTEN‐Ser380, a negative regulator of the insulin signaling cascade (Liu et al. 2014). It is worth noting that the observed increases in the most proximal brain insulin signaling mediators (e.g., pIR‐Tyr1162/Tyr1163 and pIRS‐1‐Ser636) appear to be driven as a whole group by changes observed in the fasting state, as evident by the finding that the INI had no further effects and co‐varying for fasting levels eliminated statistically significant condition effects (Figure 2). This observation is consistent with prior literature suggesting that post‐mortem (Gabbouj et al. 2019) or fasting nEV (Kapogiannis et al. 2015) insulin signaling is reduced in people with obesity and insulin resistance, and that higher insulin levels do not further activate insulin action in lean, normal glucose tolerant individuals (Hirvonen et al. 2011). While the increase in some insulin signaling proteins under fasting conditions observed in the present student are consistent with prior rodent work showing increased fasting insulin signaling post‐exercise training (Park et al. 2019; Muller et al. 2011), we did not see further elevation of any insulin signaling protein measured after INI after exercise. Recognizing our modest sample size, this could suggest that the improvements here after exercise (i.e., condition effect) is due to basal upregulation versus dynamic shifts due to INI alone. It is noteworthy, that fasting insulin levels did not change the next morning. Subsequently, it is possible that nEVs were more sensitive after exercise to the same level of fasting insulin seen during resting conditions, such that brain insulin sensitivity improved. Nonetheless, why increases in fasting nEV insulin signaling was observed in some, but not all proteins, measured in the present study is unclear. We speculate though that the lack of change in several proteins measured in this study are not likely related to increased oxidative stress and/or elevations in circulating cortisol or epinephrine since they are known to return to basal levels within a few hours after a bout of exercise and we conducted our assessments ~15 h later (Athanasiou et al. 2023; Kawamura and Muraoka 2018). However, we acknowledge that it is possible that reduced resting sympathetic tone could reduce interference with some parts of the insulin signaling pathway and not others, thereby explaining the rise seen in nEV signaling. Further work examining such potential factors are warranted.

To explore whether higher endogenous insulin levels and exercise may have interacting effects on brain insulin signaling, we provided individuals with 75 g of glucose orally and performed an OGTT. Exercise elicited a differential nEV insulin signaling biomarker response following glucose ingestion, when compared with rest, but this was largely driven by the higher fasting nEV biomarker levels. In either case, the insulin signaling proteins pIRS‐1‐Ser636, pAkt‐Ser473, and pTSC2‐Ser939 decreased nominally and pp70S6K‐Thr412 decreased significantly 60 min following glucose ingestion, compared with an increase or steady levels following rest. This finding parallels our prior work demonstrating a reduction in glucose‐stimulated pAkt/total Akt derived from nEV after 2 weeks of aerobic exercise (Malin, Battillo, et al. 2025). Notably, this interaction between exercise decreasing glucose‐stimulated nEV biomarkers in the present study compared with rest is also consistent with previous work reporting that insulin stimulates brain glucose metabolism or CBF among aging individuals with or without impaired glucose tolerance, whereas healthy individuals did not experience any effect of insulin to increase glucose metabolism (Hirvonen et al. 2011; Akintola et al. 2017). This has been interpreted to suggest brain glucose uptake is already maximal during the fasting state among lean individuals (Rebelos et al. 2019). Thus, in the present study, exercise may increase brain insulin signaling in the fasting state and decrease it in response to increased endogenous insulin levels following glucose ingestion, suggesting that the context may matter for improvements in brain insulin sensitivity under dynamic conditions. Further work is warranted to understand the exact mechanisms by which exercise modulates brain insulin signaling during the fasted, INI, and glucose‐stimulated states.

Insulin is also understood to act in the brain to promote neuronal growth and synapse formation. Here we report that exercise increased pTSC2‐Ser939, a signaling protein implicated in cell growth and division (Mizuguchi et al. 2021). The increase in pTSC2‐Ser939 may be somewhat surprising since Akt upregulation is understood to inhibit TSC2 (Inoki et al. 2002), TSC2 activation is also regulated by other proteins (e.g., extracellular signal‐regulated kinase (ERK)) not measured in the present study. In turn, it is possible that other signaling pathways after exercise played a role in mediating the observed change in TSC2, independent of Akt (Ma et al. 2005). Nonetheless, TSC2 also inhibits mTOR to modify subsequent activation of neuronal cell growth‐mediating proteins, and this parallels the lack of change we see in pmTOR‐Ser2448 (Huang et al. 2008). Interestingly, though, p70S6K is a downstream protein of mTOR that is similarly implicated in cellular processes like cell growth and neuronal proliferation and has been commonly examined as an alternative marker of mTOR activity (Kim et al. 2012). However, p70S6K is also intimately linked to IRS‐1 and one of its main kinase modulators. We note that pp70S6K‐Thr412 increased in the fasted state and declined following glucose ingestion after exercise as compared with the resting condition with a large effect size. Indeed, nutrient intake has been demonstrated to raise mTOR and p70S6K signaling, such as that observed here following rest (Deldicque et al. 2010). In concert with changes observed in the exercise condition among pIR‐Tyr1162/Tyr1163, pIRS‐1‐Ser636, pAkt‐Ser473, and pTSC2‐Ser939 may in response to glucose ingestion, exercise‐mediated pp70S6K‐Thr412 activation may decrease following glucose ingestion as brain glucose uptake may not further increase beyond the fasting condition. Future studies are needed to understand how exercise modulates protein synthesis and growth in the brain.

Insulin plays a neuroprotective role in the brain through its regulation of amyloid‐β development and tau pathology (Mullins et al. 2017). Importantly, insulin mediates both amyloid‐β deposition and tau hyperphosphorylation through, in part, its regulation of GSK‐3α and GSK‐3β. These are downstream insulin signaling proteins that are inhibited by their phosphorylation by Akt (Hernández et al. 2010). Despite the effects of exercise on pAkt‐Ser473, we did not observe a change in GSK‐3α or GSK‐3β following exercise in the present study. This finding is interesting, given that both exercise and insulin stimulation have been shown to inhibit GSK3 activation in human skeletal muscle (Markuns et al. 1999). Thus, whether chronic exercise modulates brain GSK3 activation to promote neuroprotective effects in humans awaits further investigation.

This study has limitations that warrant consideration. This was an exploratory study, and we had a relatively small sample size that may have limited our ability to discern significant interaction effects among the insulin signaling proteins. However, the similar patterns of change observed across nEV‐associated mediators at multiple levels of the cascade strengthen our conclusions that exercise has overarching effects on insulin signaling pathways. Further, the study population primarily consisted of white, middle‐aged women who were cognitively normal. Thus, our findings may not be generalizable across populations, including those with ADRD. Although the methodology for deriving nEVs employed in this manuscript has been validated (Kapogiannis et al. 2024), as it is the case with all immunoaffinity capture methodologies, recovered nEVs should be interpreted as EVs enriched for neuronal origin rather than as a purely neuronal population (Manolopoulos et al. 2025). Moreover, the time course in which INI administration may elicit changes in nEV insulin signaling is unknown. We measured nEV insulin signaling at 30 min post‐INI as prior work suggests this is when insulin is at peak action in the brain following INI administration (Morris and Burns 2012). This may have been too short to see effects on nEVs crossing the brain–blood barrier into the general circulation and further work here is warranted. Thus, these findings should be interpreted with caution, as it remains possible that nEV insulin signaling could vary over time, and more work is needed to assess nEV kinetics. Another consideration is that we are not able to identify the specific brain region from which these nEVs are released. Nonetheless, strengths of the current study include use of a within‐participant study design and that we tested the effects of a single bout of exercise, thereby minimizing potential confounding effects of chronic adaptations (e.g., fitness or body fat changes). We also examined CBF in these same individuals and report that the increase in some nEV insulin signaling biomarkers parallels rises in CBF, thereby giving some confidence that functional changes at the brain level are related to these cellular biochemical changes.

In conclusion, a single bout of aerobic exercise performed the night before increased some insulin signaling proteins classically involved in glucose metabolism as well as those implicated in neuronal cell growth and proliferation. We specifically observed that exercise may modulate some of these nEV‐derived biomarkers during the fasting and/or post‐glucose ingestion states. Therefore, further work is warranted to examine how increases in nEV‐derived insulin signaling biomarkers relate to exercise‐mediated changes in whole body insulin sensitivity as well as how chronic exercise interventions can further modulate brain insulin signaling to promote cognitive health and mitigate AD/ADRD risk.

## Author Contributions

S.K.M. conceptualized the clinical study design and hypotheses. S.K.M. and D.K. conceptualized the present nEV biomarker analytical plan and hypotheses. S.K.M., D.J.B., M.S.B., M.M. and D.K. contributed to recruitment, data collection, data analysis, and/or interpretation. D.J.B. and S.K.M. were responsible for statistical analysis. S.K.M. wrote the manuscript, and all authors provided editorial comments as well as approved the final manuscript.

## Funding

This work was supported by the Brain Health Institute, Rutgers University; National Institute of Diabetes and Digestive and Kidney Diseases, R01DK133598‐01A1 (both S.K.M). This research was also supported in part by the Intramural Research Program of the National Institutes of Health (NIH) (D.K.).

## Disclosure

The authors have nothing to report.

## Conflicts of Interest

The authors declare no conflicts of interest.

## Data Availability

The data that support the findings of this study are available on request from the corresponding author. The data are not publicly available due to privacy or ethical restrictions.
